# Rates and determinants of Rotavirus vaccine uptake among children in Italy: a cross-sectional study within the 2022 OBVIOUS* project

**DOI:** 10.1186/s12889-024-18154-0

**Published:** 2024-03-12

**Authors:** Giusy La Fauci, Giorgia Soldà, Zeno Di Valerio, Aurelia Salussolia, Marco Montalti, Francesca Scognamiglio, Angelo Capodici, Maria Pia Fantini, Heidi J. Larson, Julie Leask, Davide Gori, Jacopo Lenzi

**Affiliations:** 1 Unit of Hygiene, Public Health and Medical Statistics, Department of Biomedical and Neuromotor Sciences, University of Bologna, 40126 Bologna, Italy; 2 School of Public Health, Faculty of Medicine and Health, University of Sydney, Sydney, NSW Australia; 3 Sydney Infectious Diseases Institute, Westmead, NSW Australia; 4 Institute for Health Metrics & Evaluation (IHME), University of Washington, Seattle, WA USA; 5 London School of Hygiene and Tropical Medicine (LSHTM), London, UK

**Keywords:** Rotavirus vaccination, Vaccine uptake, Child vaccination, Immunization

## Abstract

**Introduction:**

The World Health Organization defines rotavirus as among the most severe causes of viral gastroenteritis affecting children under 5 year old. Italy and other European countries do not release disaggregated data on rotavirus vaccination coverage. This study aimed to assess the uptake and drivers of rotavirus vaccination in Italy.

**Methods:**

We administered a survey to 10,000 Italian citizens recruited via an online panel and proportionate to key demographic strata. We examined rotavirus vaccine uptake among parents whose youngest child was aged 6 weeks to 4 years, their sociodemographic characteristics, their beliefs about vaccine administration, and who recommended the rotavirus vaccination.

**Results:**

A total of 711 respondents met the inclusion criteria for the rotavirus vaccine questionnaire. The uptake was estimated at 60.3% nationwide (66.4% among mothers and 50.2% among fathers). Being a mother and living in cities/suburbs was significantly associated with a higher likelihood of vaccine uptake, while fathers were more likely to be uncertain of their children’s vaccine status. Living in Central Italy and having friends/relatives opposed to vaccination were found to be significantly associated with a lower likelihood of vaccine uptake, while parents’ education level and children’s demographics were not found to correlate with any outcomes. In 90.3% of cases, the rotavirus vaccination was recalled as being recommended by a paediatrician.

**Conclusions:**

Consistent collection of behavioural preferences and socioeconomic characteristics of recipients of rotavirus vaccine campaigns, their epidemiological information, cost-benefit, and national policy data are crucial for designing effective vaccination strategies in Italy and other European countries with similar social profiles to reach the target uptake.

**Supplementary Information:**

The online version contains supplementary material available at 10.1186/s12889-024-18154-0.

## Background

The World Health Organization (WHO) states that rotavirus is among the most severe types of viral gastroenteritis that affects children below the age of five [1]. Globally, rotavirus deaths account for about 5% of all child deaths; infants and young children constitute the most vulnerable populations to rotavirus disease because they are particularly susceptible to developing severe dehydration [1]. In Europe, the mortality rate attributed to rotavirus is low (less than 0.1 per 100,000 individuals), but estimates suggest that each year 75,000 to 150,000 children require hospitalisation due to rotavirus gastroenteritis (RVGE), and that between 150,000 and 600,000 children seek medical attention in the Emergency Department (ED) or through paediatrician consultations for RVGE-related concerns [2]. In Italy, data indicate that rotavirus infections are responsible for 17–69% of hospital admissions for acute gastroenteritis, 84% of admissions for gastroenteritis of viral origin, 61% of gastroenteritis-related admissions to the ED, and 33% of visits to the family paediatrician or general practitioner (GP) [2].

Vaccination against rotavirus has already shown its broad impact, with a 40% reduction in the prevalence of the disease recorded in countries participating in the Global Rotavirus Surveillance Network [3]. All currently available rotavirus vaccines (RVs) are live attenuated viral strains administered orally [4]. As of 2022, only 18 out of 38 European countries have implemented fully funded universal RV programmes [5], with Italy starting its national programme in 2017, aiming at ≥ 95% coverage among children in their first year of life by 2019 [6].

In low-mortality countries, the currently used vaccines have been estimated to be 86% effective against laboratory-confirmed severe RVGE among children under 12 months of age [7], with evidence of prolonged high levels of protection against severe disease in children in their third year of life [8–9]. All WHO pre-qualified anti-RV have demonstrated a good safety profile [10–11]. Following international recommendations, in Italy, RV is available free of charge to all children 6 weeks of age or older born in 2017 or later [6].

Studies from the UK, Spain, and Italy, show that a universal vaccination against rotavirus has significant clinical and economic benefits [12–14]. A study investigating the drivers of vaccination uptake in Naples (Italy) in 2018 among parents of children aged between 3 months and 3 years showed that only 15.3% reported having vaccinated their children against rotavirus infection, but that over half of the respondents would be willing to have the vaccine [15], a figure very close to the one from national coverage data for 2018 published by the Italian Ministry of Health (MoH) [7].

In the same study, when investigating parents’ beliefs, results showed that more than half (56.4%) were worried that their children could contract rotavirus gastroenteritis and almost two-thirds (60.8%) considered the rotavirus vaccine important for their child. Factors associated with higher vaccination rates included considering rotavirus dangerous for their children, having a positive perception of the vaccine’s effectiveness, and receiving information from physicians [15]. A survey among 437 parents in Italy showed that vaccine hesitancy was more prevalent in individuals who expressed concerns about vaccine safety, the possibility of serious side effects, doubts about vaccine efficacy, those who delayed or refused vaccines for their children, and those who were uncertain about their paediatrician’s recommendations [16].

Overall, however, there is limited data on the causes of low uptake in Italy and other European countries concerning RV [17].

This study aims to collect more data on vaccine uptake and associated factors in parents and to understand the main drivers of RV. Such information will assist public health authorities and paediatricians to organise a coherent and impactful vaccination strategy aimed at increasing vaccine uptake.

## Methods

### Study design and main objectives

We conducted a cross-sectional study investigating the uptake and attitudes of Italian citizens towards five vaccines made available free of charge to specific categories by the Italian National Health Service: Rotavirus, Pneumococcal, Human Papillomavirus, influenza, and Herpes Zoster Virus. We administered a questionnaire to a representative sample of 10,000 Italian residents. This study focuses on respondents to the section of the RV questionnaire. The main objective of this study was to assess RV uptake stratified by population targets of national vaccination campaigns and drivers of vaccination.

### Questionnaire

The extended version of the questionnaire, designed to be completed in ~ 10 min, was divided into six sections investigating (Sect. 1) demographic data and (Sects. 2–6) uptake and attitudes towards RV and the other four above-mentioned vaccinations among population targets of vaccination campaigns. The survey was developed based on the WHO Behavioural and Social Drivers (BeSD) framework [18], which outlined validated survey tools aimed at assessing the main drivers of COVID-19 vaccination and routine vaccination in children. We selected the questions to assess demographic information and drivers of vaccination [19]. All respondents accessed Sect. 1, while only respondents with specific characteristics (age groups, gender, clinical conditions, body mass index, professions, or combinations thereof) had access to Sects. 2–6 on specific vaccines, in accordance with the population-target definitions provided by the 2017–2019 Italian National Vaccination Plan (*Piano nazionale prevenzione vaccinale* [PNPV]) [6]. An English version of the questionnaire for RV respondents is available in Additional file 1 in the Supplementary Material.

### Inclusion criteria for RV section

Access to the section of the questionnaire focusing on RV was granted to adult respondents who declared to be a parents whose youngest child was between 6 weeks and 4 years old. In the case of a parent with more than one child, the questions were directed at the youngest child’s vaccination status. The lower age limit was defined following international and Italian MoH’s recommendations about the earliest time vaccination should be administered, while the upper age limit was defined by the birth cohorts that were first included in Italy’s PNPV [6] against rotavirus, that is, children born in 2017 or later. The first two cohorts of children born in 2017 and 2018 (5 to 6-year-olds) were left out in order to minimise recall bias and the underestimation effect of likely less-than-optimal vaccine uptake during the very first years of the campaign.

### Data collection

The survey was conducted between April 11 and May 29, 2022, using computer-assisted web interviewing (CAWI). The professional online panel provider Dynata recruited a national sample of 10,000 Italian respondents aged 18 and older using a stratified sampling based on proportionate allocation by first-level Nomenclature of territorial units for statistics (NUTS) statistical region of residence (Northwest, Northeast, Center, South, and Islands), gender, and age group (18–24, 25–34, 35–44, 45–54, 55–64, and ≥ 65 years). Prior to the full implementation of the survey, cognitive testing was conducted to assess its effectiveness and gather feedback. The feedback received from the testing phase was used to make revisions and improvements to the questionnaire. The first section of the survey was based on the WHO Behavioural and Social Drivers (BeSD) survey, which provided a framework for investigating the behavioural and social factors influencing vaccination decisions. Post-stratification confirmed that non-response to the survey in some strata of Italy’s adult target population had no substantial effect on the study estimates [19]. For this reason, adjustment of sampling weights was deemed unnecessary to be performed on the targeted subsample of respondents for RV. Collected data were managed under the General Data Protection Regulation (GDPR) of the European Union (EU), which ensures the protection and privacy of personal data of individuals within the EU. Furthermore, data collection, storage, and processing were conducted per applicable laws and guidelines in Italy.

### Statistical analysis

All variables were summarised as counts and percentages and were stratified by first-level NUTS statistical region of residence and parental gender. Data were visualised with the aid of thematic maps with pie charts. Multivariable multinomial logistic regression analysis was carried out to examine the drivers (determinants) of RV vaccine uptake, which was considered as a three-category nominal outcome (“Yes” vs. “No” vs. “Not sure”). In keeping with the increasing vaccination model proposed by the BeSD Expert Working Group [18], the covariates which we decided to include in the regression model as potential drivers of vaccine uptake were social processes (friends and family’s views on vaccination, gender) and relevant sociodemographic determinants measured at the parent and child level (age group, statistical region of residence, place of residence degree of urbanisation, and educational attainment). The effect of covariates was assessed by examining the marginal effect of changing their values on the average predicted probability of observing each outcome. The marginal effect was computed as a discrete difference in probabilities (*Δ*), with 95% confidence intervals (CIs) obtained with the delta method. Covariate categories occurring in < 5% of the sample were combined with adjacent lower or upper classes to improve the stability and efficiency of regression estimates. The Small–Hsiao test of independence of irrelevant alternatives (IIA) did not indicate the need for alternative model specifications in which binary logit coefficients do not converge in probability to the same values as the multinomial logit coefficients, such as the nested logit model. Lastly, in order to check for the presence of moderators, that is, covariates *Z* that change the effect of other independent variables *X* on vaccine uptake, we included pairwise interaction terms *Z*×*X* in the model one at a time and tested their statistical significance with the likelihood-ratio test. To control for type I errors related to multiple testing, the significance level for interactions was set at 0.01. All analyses were conducted using Stata software, version 17. No multicollinearity issues were found in regression analysis, that is, the variance inflation factor was < 5 and the condition index was < 10 for each covariate.

## Results

### Sociodemographic characteristics of the sample

Nationwide, a total of 711 respondents with children fulfilled the criteria for access to the RV section. Out of the study sample, 433 (62.3%) were female, 267 (37.6%) male, and one (0.1%) non-binary. Mean age was 36.3 ± 8.2 years, while 40.8% lived in cities, 45.9% in towns or suburbs, and 13.4% in rural areas. Regarding educational attainment, 55.0% of the parents had a high school diploma, 27.0% an academic degree, 10.4% a postgraduate degree or doctorate, and 7.6% less than a high school diploma. Eighty-two-point 1% stated that they lived as a couple, 9.0% with their parents or relatives, and 2.5% alone. In addition, 48.8% of the respondents reported that they were able to pay for their daily expenses with some difficulty, 36.1% quite easily, 9.8% with great difficulty, and 5.2% easily. All general information about the sociodemographic characteristics of the sample is shown in Table 1.

**Table 1:** Sociodemographic characteristics of the parents constituting the study sample, overall and by NUTS statistical region

Characteristic	Italy	Northwestern Italy	Northeastern Italy	Central Italy	Southern Italy	Insular Italy
	(*n* = 711)	(*n* = 194)	(*n* = 105)	(*n* = 152)	(*n* = 184)	(*n* = 76)
Gender						
Male	267 (37.6%)	73 (37.6%)	34 (32.4%)	58 (38.2%)	77 (41.8%)	25 (32.9%)
Female	443 (62.3%)	120 (61.9%)	71 (67.6%)	94 (61.8%)	107 (58.2%)	51 (67.1%)
Non-binary	1 (0.1%)	1 (0.5%)	0 (0.0%)	0 (0.0%)	0 (0.0%)	0 (0.0%)
Age group, y						
18–24	43 (6.0%)	8 (4.1%)	9 (8.6%)	6 (3.9%)	15 (8.2%)	5 (6.6%)
25–34	242 (34.0%)	63 (32.5%)	44 (41.9%)	53 (34.9%)	59 (32.1%)	23 (30.3%)
35–44	336 (47.3%)	95 (49.0%)	35 (33.3%)	76 (50.0%)	89 (48.4%)	41 (53.9%)
45–54	68 (9.6%)	23 (11.9%)	11 (10.5%)	13 (8.6%)	16 (8.7%)	5 (6.6%)
55–64	14 (2.0%)	2 (1.0%)	4 (3.8%)	3 (2.0%)	3 (1.6%)	2 (2.6%)
≥ 65	8 (1.1%)	3 (1.5%)	2 (1.9%)	1 (0.7%)	2 (1.1%)	0 (0.0%)
Place of residence degree of urbanization^*^						
City (densely populated area)	290 (40.8%)	97 (50.0%)	33 (31.4%)	62 (40.8%)	75 (40.8%)	23 (30.3%)
Town or suburb (intermediate density area)	326 (45.9%)	79 (40.7%)	56 (53.3%)	69 (45.4%)	79 (42.9%)	43 (56.6%)
Rural area (thinly populated area)	95 (13.4%)	18 (9.3%)	16 (15.2%)	21 (13.8%)	30 (16.3%)	10 (13.2%)
Educational attainment						
Less than high school diploma	54 (7.6%)	15 (7.7%)	7 (6.7%)	17 (11.2%)	7 (3.8%)	8 (10.5%)
High school diploma	391 (55.0%)	117 (60.3%)	53 (50.5%)	78 (51.3%)	91 (49.5%)	52 (68.4%)
Academic degree	192 (27.0%)	40 (20.6%)	30 (28.6%)	42 (27.6%)	67 (36.4%)	13 (17.1%)
Post-graduate/Doctorate degree	74 (10.4%)	22 (11.3%)	15 (14.3%)	15 (9.9%)	19 (10.3%)	3 (3.9%)
Occupation						
Teacher	41 (5.8%)	11 (5.7%)	6 (5.7%)	8 (5.3%)	11 (6.0%)	5 (6.6%)
Student	25 (3.5%)	6 (3.1%)	3 (2.9%)	3 (2.0%)	11 (6.0%)	2 (2.6%)
Healthcare worker (excl. medical doctor)	23 (3.2%)	5 (2.6%)	5 (4.8%)	6 (3.9%)	6 (3.3%)	1 (1.3%)
Law enforcement member	12 (1.7%)	2 (1.0%)	2 (1.9%)	2 (1.3%)	4 (2.2%)	2 (2.6%)
Medical doctor	10 (1.4%)	1 (0.5%)	2 (1.9%)	3 (2.0%)	2 (1.1%)	2 (2.6%)
Other occupation	436 (61.3%)	131 (67.5%)	65 (61.9%)	97 (63.8%)	105 (57.1%)	38 (50.0%)
Unemployed	156 (21.9%)	36 (18.6%)	20 (19.0%)	31 (20.4%)	44 (23.9%)	25 (32.9%)
Retired	8 (1.1%)	2 (1.0%)	2 (1.9%)	2 (1.3%)	1 (0.5%)	1 (1.3%)
Household composition						
Alone	18 (2.5%)	4 (2.1%)	3 (2.9%)	3 (2.0%)	6 (3.3%)	2 (2.6%)
Couple	584 (82.1%)	166 (85.6%)	83 (79.0%)	128 (84.2%)	146 (79.3%)	61 (80.3%)
With parents/family	64 (9.0%)	10 (5.2%)	11 (10.5%)	16 (10.5%)	19 (10.3%)	8 (10.5%)
Other	45 (6.3%)	14 (7.2%)	8 (7.6%)	5 (3.3%)	13 (7.1%)	5 (6.6%)
Able to pay for things needed in life						
With great difficulty	70 (9.8%)	17 (8.8%)	12 (11.4%)	14 (9.2%)	15 (8.2%)	12 (15.8%)
With some difficulty	347 (48.8%)	83 (42.8%)	55 (52.4%)	68 (44.7%)	100 (54.3%)	41 (53.9%)
Quite easily	257 (36.1%)	80 (41.2%)	28 (26.7%)	67 (44.1%)	62 (33.7%)	20 (26.3%)
Easily	37 (5.2%)	14 (7.2%)	10 (9.5%)	3 (2.0%)	7 (3.8%)	3 (3.9%)

^*^According to the Eurostat Degree of Urbanisation (DEGURBA) classification system

*Notes*: Northwestern Italy includes the regions of Piedmont, Aosta Valley, Lombardy, and Liguria; Northeastern Italy includes the regions of Trentino-South Tyrol, Veneto, Friuli-Venezia Giulia, and Emilia-Romagna; Central Italy includes the regions of Tuscany, Umbria, Marche, and Lazio; Southern Italy includes the regions of Abruzzo, Molise, Campania, Apulia, Basilicata, and Calabria; Insular Italy includes the regions of Sicily and Sardinia

### Rotavirus vaccination rates

**Fig. 1:**
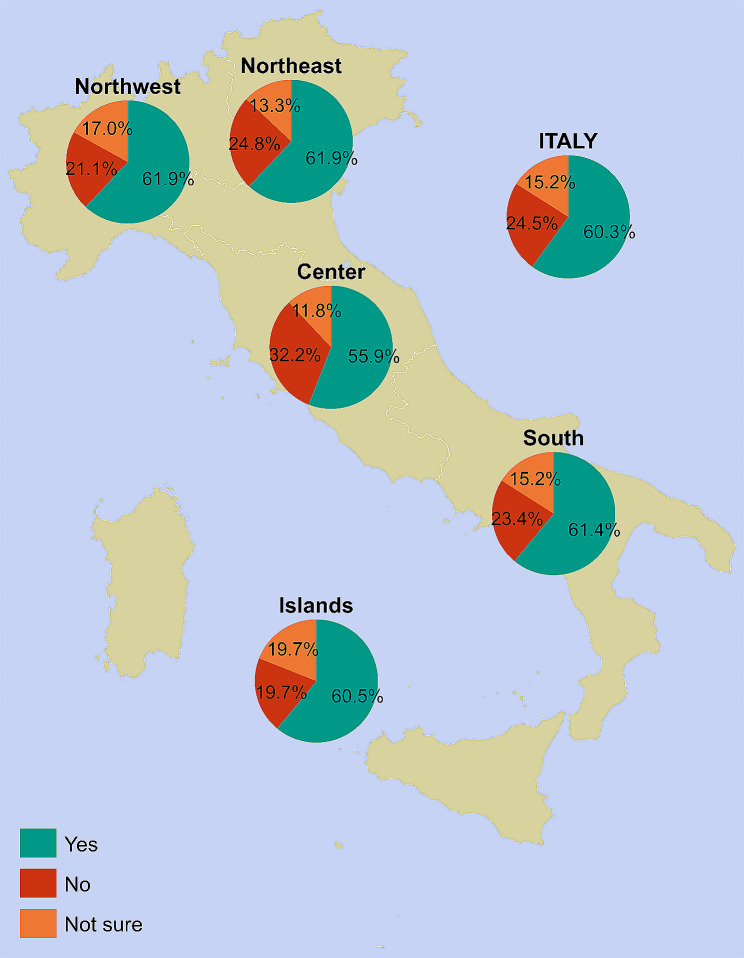
Rotavirus vaccine uptake among children between 6 weeks and 4 years of age (*n* = 711), overall and by NUTS statistical region. *Notes*: Northwestern Italy includes the regions of Piedmont, Aosta Valley, Lombardy, and Liguria; Northeastern Italy includes the regions of Trentino-South Tyrol, Veneto, Friuli-Venezia Giulia, and Emilia-Romagna; Central Italy includes the regions of Tuscany, Umbria, Marche, and Lazio; Southern Italy includes the regions of Abruzzo, Molise, Campania, Apulia, Basilicata, and Calabria; Insular Italy includes the regions of Sicily and Sardinia. *NUTS*, Nomenclature of Territorial Units for Statistics

Overall, self-reported RV uptake was 60.3%, ranging from 55.9% in Central Italy to 61.9% in both Northwestern and Northeastern Italy (Fig. 1). We also found that, at the national level, 15.2% of parents were not sure of their child’s vaccination status, ranging from 11.8% in Central Italy to 19.7% in Insular Italy (Fig. 1).

**Fig. 2:**
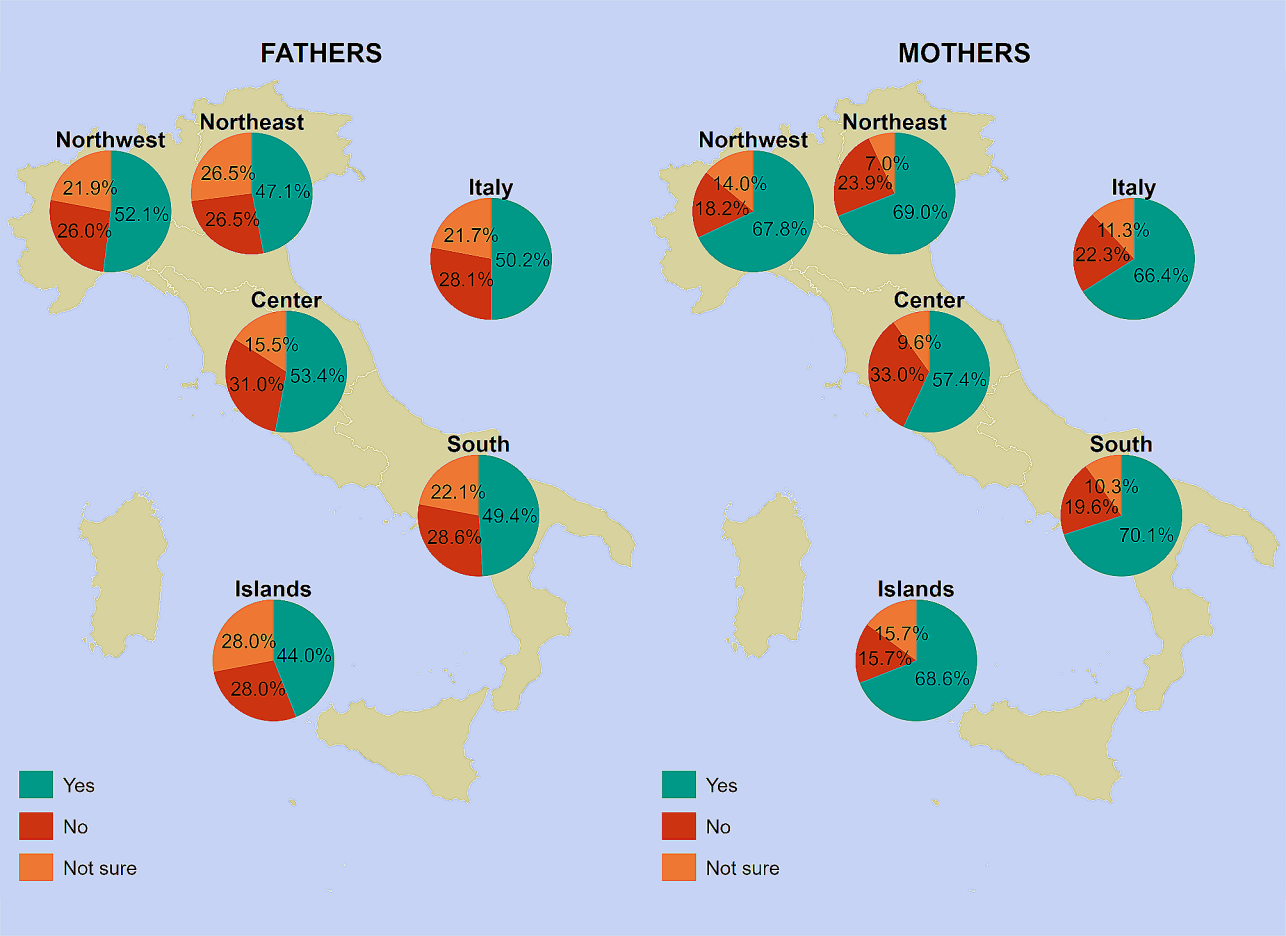
Rotavirus vaccine uptake among children between 6 weeks and 4 years of age as reported by fathers (*n* = 267) vs. mothers (*n* = 444), overall and by NUTS statistical region. *Notes*: Mothers include non-binary persons. Northwestern Italy includes the regions of Piedmont, Aosta Valley, Lombardy, and Liguria; Northeastern Italy includes the regions of Trentino-South Tyrol, Veneto, Friuli-Venezia Giulia, and Emilia-Romagna; Central Italy includes the regions of Tuscany, Umbria, Marche, and Lazio; Southern Italy includes the regions of Abruzzo, Molise, Campania, Apulia, Basilicata, and Calabria; Insular Italy includes the regions of Sicily and Sardinia. *NUTS*, Nomenclature of Territorial Units for Statistics

When the national data were disaggregated by parental gender (Fig. 2), we found that RV uptake reported by mothers and fathers was 66.4% and 50.2%, respectively. Uptake rates were consistently higher among mothers than among fathers in all geographical areas (Fig. 2): according to gender and territory combined, the highest reported RV uptake was 70.1% for mothers in the South, while the lowest reported RV uptake was 44.0% for fathers in the Islands. Figure 2 also shows the percentages of respondents who did not vaccinate their children and were not sure of their children’s vaccination status, by parental gender and NUTS statistical region.

### General information about vaccines

Within our sample, 35.6% of the respondents preferred to have their vaccinations in a vaccine hub, 23.2% at the hospital, 22.9% at their family paediatrician or GP, and the remaining 18.4% in living environments (8.2% home, 6.8% pharmacy, and 3.4% workplace). The highest preference for vaccine hubs was reported by participants from Southern Italy (40.2%), and the lowest by participants from Insular Italy (27.6%).

Furthermore, 7.3% of the sample reported that their friends and relatives had an unfavourable or very unfavourable opinion on vaccination, compared to 51.0% who had friends and relatives with a favourable or very favourable opinion. The highest rate of participants who had friends and relatives with an unfavourable or very unfavourable opinion about vaccines was observed in the Northeast (13.4%), and the lowest in the Islands (3.9%). All general information about vaccines described in this subsection is summarised in Table S1 in the Supplementary Materials.

### Parent-reported information on their youngest children

Among the respondents’ youngest children (*n* = 711), 45.4% were girls and 54.6% were boys. In general, the mean age was 2.0 ± 1.1 years. Table 2 shows the demographic characteristics of respondents’ youngest children, overall and by NUTS statistical region. Regarding those in charge of the child’s vaccinations, mothers answered that 55.3% of them were the main decision-makers, compared to 37.8% of fathers (Table S2 in the Supplementary Materials). Among the children with known vaccine uptake (*n* = 603), 322 (53.4%) attended nursery school or other children’s communities.

**Table 2:** Information about the youngest children of respondents, overall and by NUTS statistical region

Characteristic	Italy	Northwestern Italy	Northeastern Italy	Central Italy	Southern Italy	Insular Italy
	(*n* = 711)	(*n* = 194)	(*n* = 105)	(*n* = 152)	(*n* = 184)	(*n* = 76)
Gender						
Male	388 (54.6%)	97 (50.0%)	49 (46.7%)	87 (57.2%)	111 (60.3%)	44 (57.9%)
Female	323 (45.4%)	97 (50.0%)	56 (53.3%)	65 (42.8%)	73 (39.7%)	32 (42.1%)
Age group						
6 to 8 weeks	11 (1.5%)	4 (2.1%)	1 (1.0%)	3 (2.0%)	2 (1.1%)	1 (1.3%)
9 weeks to 5 months	83 (11.7%)	27 (13.9%)	19 (18.1%)	17 (11.2%)	14 (7.6%)	6 (7.9%)
6 months 3 years	617 (86.8%)	163 (84.0%)	85 (81.0%)	132 (86.8%)	168 (91.3%)	69 (90.8%)
Who takes charge of the child’s vaccinations						
Mostly myself	346 (48.7%)	88 (45.4%)	52 (49.5%)	70 (46.1%)	100 (54.3%)	36 (47.4%)
Mostly my partner	72 (10.1%)	15 (7.7%)	10 (9.5%)	13 (8.6%)	22 (12.0%)	12 (15.8%)
Equally myself and my partner	293 (41.2%)	91 (46.9%)	43 (41.0%)	69 (45.4%)	62 (33.7%)	28 (36.8%)

*Notes*: Northwestern Italy includes the regions of Piedmont, Aosta Valley, Lombardy, and Liguria; Northeastern Italy includes the regions of Trentino-South Tyrol, Veneto, Friuli-Venezia Giulia, and Emilia-Romagna; Central Italy includes the regions of Tuscany, Umbria, Marche, and Lazio; Southern Italy includes the regions of Abruzzo, Molise, Campania, Apulia, Basilicata, and Calabria; Insular Italy includes the regions of Sicily and Sardinia. *NUTS*, Nomenclature of Territorial Units for Statistics

### Determinants of RV uptake and knowledge about youngest child’s vaccination status

As shown in Table 3, the multivariable multinomial logistic regression analysis of vaccine uptake over parental characteristics revealed that being aged 45 years or older (age ≥ 45: 36.5%; age 18–34: 26.8%; *Δ* = 10.1, 95% CI = 0.0 to 20.2), living in Central Italy (Center: 32.7%; Northwest: 21. 1%; *Δ* = 11.6, 95% CI = 2.3 to 20.9), and having friends or relatives against vaccination (“quite to very unfavourable”: 35.0%; “very favourable”: 21.8%; *Δ* = 13.2, 95% CI = 2.5 to 23.9) were associated with the child not having received the RV vaccine. Conversely, we found that mothers (female: 65.2%; male: 52.3%; *Δ* = 12.9, 95% CI = 5.5 to 20.3), respondents aged between 35 and 44 years (age 35–44: 67.1%; age 18–34: 57.1%; *Δ* = 10.0, 95% CI = 2.5 to 17.5), and living in towns or suburbs (town or suburbs: 64.2%; city: 55.4%; *Δ* = 8.8, 95% CI = 1.2 to 16.4) had a higher probability of RV vaccine uptake.

**Table 3:** Results of multinomial multivariable logistic regression analysis: determinants of rotavirus vaccine uptake, non-uptake of rotavirus vaccine, and uncertainty of vaccination status of the youngest child among the respondents (*n* = 711)

	Yes			No			Not sure		
Characteristic	Predicted	Discrete difference (*Δ*)		Predicted	Discrete difference (*Δ*)		Predicted	Discrete difference (*Δ*)	
	Probability	Estimate	95% CI	Probability	Estimate	95% CI	Probability	Estimate	95% CI
Parent’s gender									
Male	52.2%	Ref.		26.3%	Ref.		21.6%	Ref.	
Female†	65.3%	13.1*	5.6, 20.5	23.4%	−2.8	−9.4, 3.7	11.3%	−10.3*	−16.1, − 4.4
Parent’s age group, y									
18–34	57.3%	Ref.		26.8%	Ref.		15.9%	Ref.	
35–44	67.2%	9.9*	2.4, 17.4	19.4%	−7.3*	−13.9, − 0.8	13.4%	−2.5	−8.2, 3.1
≥ 45	44.1%	−13.2*	−24.9, − 1.5	36.8%	10.1*	0.0, 20.2	19.1%	3.2	−5.8, 12.1
NUTS statistical region									
Northwestern Italy	61.9%	Ref.		21.0%	Ref.		17.1%	Ref.	
Northeastern Italy	63.6%	1.7	−9.4, 12.8	23.5%	2.5	−7.2, 12.2	12.9%	−4.2	−12.4, 4.0
Central Italy	55.7%	−6.2	−16.3, 3.9	32.7%	11.7*	2.4, 20.9	11.6%	−5.5	−12.7, 1.8
Southern Italy	62.2%	0.3	−9.3, 9.9	22.6%	1.6	−6.7, 9.8	15.2%	−1.9	−9.3, 5.5
Insular Italy	56.7%	−5.1	−17.9, 7.6	22.6%	1.6	−9.6, 12.9	20.6%	3.5	−7.0, 14.1
Degree of urbanization‡									
City	54.9%	Ref.		27.9%	Ref.		17.1%	Ref.	
Town or suburb	64.6%	9.7*	2.1, 17.3	22.7%	−5.3	−12.1, 1.5	12.7%	−4.4	−10.1, 1.2
Rural area	62.4%	7.5	−3.5, 18.6	20.0%	−7.9	−17.4, 1.5	17.6%	0.4	−8.2, 9.1
Parent’s educational attainment									
Post-graduate/Doctorate degree	52.8%	Ref.		33.1%	Ref.		14.1%	Ref.	
Academic degree	56.2%	3.4	−9.4, 16.2	28.8%	−4.3	−16.4, 7.8	15.1%	0.9	−8.5, 10.3
High school diploma	64.5%	11.7	−0.3, 23.7	21.3%	−11.8	−23.5, 0.0	14.2%	0.1	−8.7, 8.8
Less than high school diploma	56.5%	3.7	−13.2, 20.6	19.1%	−13.9	−28.6, 0.7	24.4%	10.2	−3.6, 24.0
Child’s gender									
Male	62.7%	Ref.		23.3%	Ref.		14.0%	Ref.	
Female	57.5%	−5.2	−12.2, 1.8	25.8%	2.5	−3.7, 8.8	16.7%	2.7	−2.6, 8.0
Child’s age group									
6 wk to 5 m	59.4%	Ref.		20.8%	Ref.		19.8%	Ref.	
6 m to 3 y	60.4%	1.0	−9.3, 11.3	25.1%	4.3	−4.2, 12.9	14.5%	−5.3	−13.7, 3.0
Dear ones’ views on vaccination in general									
Very favorable	68.2%	Ref.		21.6%	Ref.		10.2%	Ref.	
Favorable	66.1%	−2.1	−11.5, 7.3	19.0%	−2.5	−10.7, 5.6	14.8%	4.6	−2.0, 11.3
Quite favorable	54.2%	−14.0*	−23.3, − 4.7	25.8%	4.3	−4.1, 12.6	19.9%	9.7*	2.8, 16.6
Quite to very unfavorable	51.2%	−17.0*	−28.5, − 5.6	35.5%	13.9*	3.2, 24.7	13.3%	3.1	−4.7, 10.8

*P-value ≤ 0.05, that is, *Δ* significantly ≠ 0

†Including non-binary persons

‡According to the Eurostat Degree of Urbanisation (DEGURBA) classification system

NUTS, Nomenclature of Territorial Units for Statistics

Fathers were more likely to be unsure of their children’s RV status (mothers: 11.3%; fathers: 21.6%; *Δ* = -10.2, 95% CI = -16.1 to -4.4). Educational attainment and child demographics were not related to any of the study outcomes. The analysis of possible interaction effects did not reveal the presence of any significant moderators among covariates.

No differences in RV uptake were found between children attending vs. not attending nursery schools or other children’s communities (71.7% vs. 70.5%; Pearson χ² = 0.12, *P*-value = 0.73). There were discrepancies across NUTS statistical regions, but the estimates are perturbed by small denominators ( Figure S1 in the Supplementary Material).

**Fig. 3:**
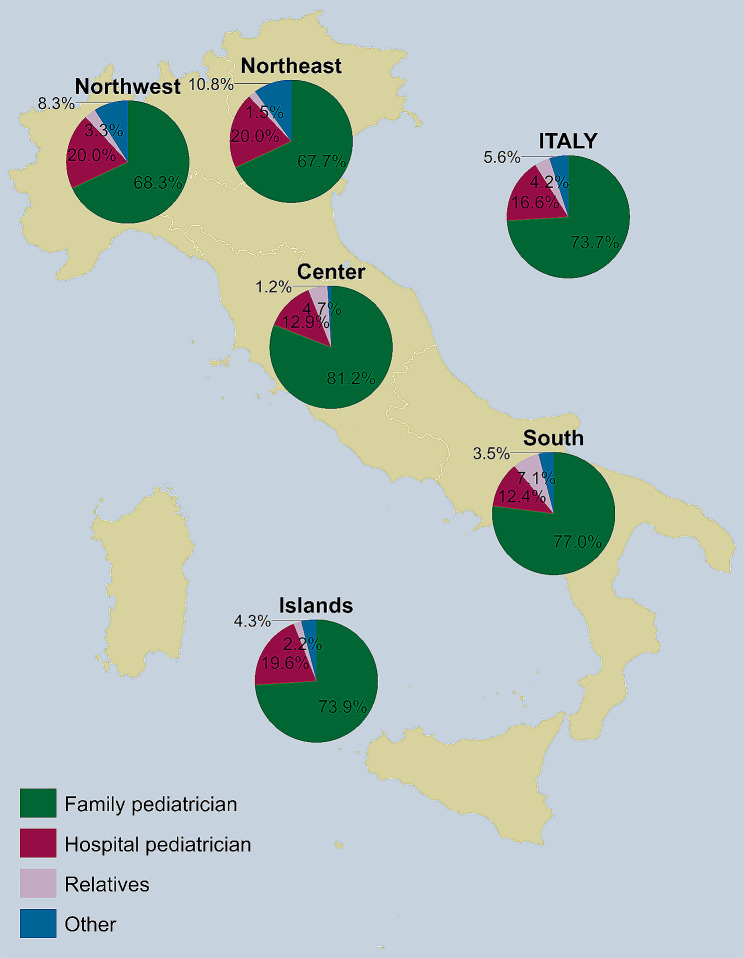
Persons who recommended the parents that children get rotavirus vaccine (*n* = 429), overall and by NUTS statistical region. *Notes*: Northwestern Italy includes the regions of Piedmont, Aosta Valley, Lombardy, and Liguria; Northeastern Italy includes the regions of Trentino-South Tyrol, Veneto, Friuli-Venezia Giulia, and Emilia-Romagna; Central Italy includes the regions of Tuscany, Umbria, Marche, and Lazio; Southern Italy includes the regions of Abruzzo, Molise, Campania, Apulia, Basilicata, and Calabria; Insular Italy includes the regions of Sicily and Sardinia. *NUTS*, Nomenclature of Territorial Units for Statistics

Lastly, regarding who recommended the RV, Fig. 3 illustrates that among the 429 children who had the RV, in 73.7% of cases the vaccine was recommended by the family paediatrician, in 16.6% by the hospital paediatrician, in 4.2% by relatives, and in 5.6% by others.

## Discussion

The RV uptake of 60.3% observed in this study aligns with the Italian MoH’s reported coverage figure of 62.8% forthe 2018 cohort [20]. The slightly higher figure for this cohort as compared to our 2022 estimate may be due to lingering disruptions in routine vaccination programmes during the COVID-19 pandemic and parent concerns about going to health services. This figure also highlights the difficulties in attaining the goals initially set by the PNPV 2017–2021, which aimed at having at least 95% of newborns vaccinated against RV by 2019 [6].

Our data reveal regional differences in vaccine adoption in Italy. Central Italy has lower RV uptake rates than the southern and northern regions. This finding is supported by multivariable (adjusted) analysis, which shows a significant association between residing in Central Italy and lower RV adherence (Table 3). The delay in implementing free and open RV campaigns in central Italian regions such as Umbria, Marche, and Lazio may explain this disparity, while some southern regions had already offered the vaccine before it became a statewide standard in 2017 [7]. Studies show shows how the delay in active and free RV offer has had an impact on hospitalizations for rotavirus gastroenteritis even in a high-income country like Italy. In fact, until 2018 there was an increasing trend of rotavirus gastroenteritis hospitalizations in regions where the vaccination had only been offered since 2017 and vaccination coverage levels were suboptimal [21]. At the same time, the trend in rotavirus hospitalizations had a decreasing trend over the same periods in a similar study conducted in another Italian region where vaccination had been introduced as early as 2013 [22].

The RV uptake estimated in our study at the national level is lower than the uptake in the United States and many European countries [23], highlighting the need to analyse the main determinants of uptake and non-uptake in Italy.

With respect to the characteristics of respondents, parental gender seemed to play a relevant role in attitudes toward RV, since vaccine uptake was higher in female parents than in male parents. In our sample, mothers were also 1.5 times more likely than fathers to be the main decision-makers regarding their children’s vaccinations, and, confirming this, fathers were found to be more likely to be unsure of their children’s RV status. These data confirm that mothers remain the most involved decision-makers regarding children’s health, including vaccinations [24]. This holds especially true in Italy, where parental care and family health remain mainly women’s responsibilities [25]. This also confirms mothers as the best target of informational campaigns aimed at increasing vaccination uptake in children, and a more reliable source of information when investigating children’s vaccinations.

Furthermore, in this study, the parent’s educational attainment did not correlate with any of the study outcomes. Currently, there is no concordance in the literature on the role of parent’s educational attainment in their children’s vaccinations [26–27]. According to recent evidence, factors more specific than parent’s educational attainment, such as health literacy or vaccination literacy, may have a greater impact on children’s vaccination uptake [28–29].

This nationwide survey also shows that, among parents of children vaccinated against rotavirus, family paediatricians were the most likely to recommend the RV. Family paediatricians have a central role in Italy in managing health issues of children and adolescents, similar to the role of GPs for adults. This result confirms the importance of health professionals in providing information on vaccines and their accessibility and addressing safety concerns, identifying them as the most trusted figures when seeking counselling on health matters [30–31].

Furthermore, in our sample, having friends and relatives with negative opinions on vaccination was associated with low uptake, especially in Central Italian regions. This underlines the importance of the social environment in making decisions on vaccination: certain attitudes and behaviours may be harder to influence because of social structures and networks that prove resistant to external inputs. Approaches that are not necessarily focused only on individuals but on their complex social interactions may prove more useful in effectively delivering information and providing counsel [24].

Practical issues have a key role in influencing vaccine uptake: this prompted us to investigate multiple potential barriers to vaccination. Among these, no clear preference on where respondents might prefer to receive vaccinations emerged, with approximately an equal share between vaccine hubs, hospitals, and GPs. This suggests the possibility that offering vaccinations in several locations might be necessary to accommodate a wide range of needs. Data on preferences also confirm that primary care practices remain an important reference point in providing healthcare, at least in the Italian system, and thus a useful starting point for tackling VH and reinforces what has already been described in the literature [30].

To our knowledge, our study is the first nationwide cross-sectional study on RV uptake in a European country to provide an up-to-date overview of adoption and barriers to RV vaccination. In fact, according to WHO data, RV coverage in Europe is gradually increasing over time while reaching a European average of 34% in 2021 [23].

Studying drivers of RV uptake in European countries is crucial for developing effective vaccination programmes and overcoming specific barriers in each territory. Additionally, more accurate data collection and analysis of rotavirus-related direct and indirect costs is needed to understand the impact of the vaccine on a global and European scale, whose cost-benefit ratio is often underestimated [32]. In this regard, thanks to vaccination there seems to be a reduction in care costs and work absenteeism [33–34], important aspects to consider to properly inform policymakers. It must also be noted that evidence from other Rotavirus vaccination campaigns in countries such as Finland and the United Kingdom appears to favour a different approach in the organisation of Rotavirus vaccination campaigns than the one chosen by the Italian government, that is, a timed strategy that prioritises mass vaccination before the seasonal winter epidemic [35]. If such an approach were to become the norm in Italy, the relative weight effect of uptake figures on the long-term benefits of vaccination may change, as could the importance of single predictors found by this study.

This study must be considered also in light of its limitations. First, the cross-sectional design prevents causal inferences. Second, the web-based survey administration may introduce selection bias, due to web accessibility and digital expertise, which may lead to limitations in generalisability. Third, the study data consisted of self-reported responses, which may lead to information bias; however, we think that limiting the study to parents of children aged 4 years or younger may have mitigated recall bias. Lastly, the overall study sample of 711 included fathers and, more generally, parents uncertain about their children’s vaccination status. The resulting figure of 60.3% might thus underestimate the actual RV uptake in Italy’s child population. Quantifying the extent of this bias is challenging and implies unsubstantiated assumptions about the strata of the population not reached by the survey. However, discarding fathers and hypothesising that the uptake among the 11.3% of mothers who were unable to provide a definite answer was identical to that observed in the other respondents, a less conservative, yet likely speculative alternative to the overall estimate of 60.3% is represented by 74.9%, calculated as 0.664 × 100/(1–0.113).

## Conclusions

Being relatively recently introduced and covering a very limited population age group, data and studies on RV are quite scarce when compared to other vaccines, leading to decision-making processes that are not as evidence-based as others. The evidence produced so far needs to be updated since it was created some years ago to lead governments to introduce RV to all infants [33]; therefore, there is a need for more data measuring the effect and impact of this vaccination on a European and Global scale. We believe that consistent epidemiological data collected regularly are key to informing future research on this topic and, therefore, policymaking.

The findings from our study provide a starting point for decision-makers in Italy and other European countries with similar social profiles to design effective vaccination strategies that address the preferences of at-risk groups and overcome the barriers associated with vaccine uptake.

### Electronic supplementary material

Below is the link to the electronic supplementary material.

Supplementary Material 1

## Data Availability

Data can be made available upon request and at discretion of the OBVIOUS board by contacting the corresponding author.
