# Supplementary material for: Rates and determinants of Rotavirus vaccine uptake among children in Italy: a cross-sectional study within the 2022 OBVIOUS* project
Source: BMC Public Health. 2024 Mar 12;24:770. doi: 10.1186/s12889-024-18154-0 (PMC10935934; doi:10.1186/s12889-024-18154-0)
Supplement: Supplementary file 1 — Supplementary Material 1 [file 12889_2024_18154_MOESM1_ESM.pdf]

Proposta per Università Degli Studi di Bologna
mercoledì 16 febbraio 2022

ITA vaccini - DEF

QUO-1425763-Y3H7

Tipo di servizio: End to End

Tipo di progetto: Standard

Descrizione del progetto

Prezzi

Costo totale: € 25.200,00

**Livello riepilogativo**	**Completati**	**CPI** [EUR]	**Costo totale** [EUR]
Tutti i Paesi e i servizi aggiuntivi	10000	€ 2,52	€ 25.200,00
		**Prezzo totale:**	**€ 25.200,00**

**Paese**	**Completati**	**CPI** [EUR]	**Costo totale** [EUR]
Online Completes Italy 80,0% 15 min	10000	€ 2,20	€ 22.000,00
		**Costo totale del campione:**	**€ 22.000,00**

**Servizi aggiuntivi**	**Costo totale** [EUR]
Online Data Processing (Excel Data File)	€ 350,00
Online Data Processing (Excel Data File)	€ 350,00
Online Standard Program & Host	€ 2.500,00
**Totale servizi aggiuntivi:**	**€ 3.200,00**

Condizioni dell’ordine

Salvo altrimenti specificato in un accordo scritto stipulato tra il Cliente e Dynata, le condizioni dell’ordine con Dynata sono quelle riportate di seguito.

1. **Informazioni generali**. Le informazioni indicative relative a prezzi e tempistiche contenute nel preventivo sono state determinate in funzione del campo di applicazione del progetto, dei materiali del sondaggio, delle informazioni e delle caratteristiche specifiche del progetto che il Cliente fornisce a Dynata alla data del presente preventivo. Tutte le stime su prezzi e tempistiche possono essere soggette a modifica in funzione delle caratteristiche tecniche definitive specifiche del progetto e/o dei materiali del sondaggio trasmessi dal Cliente. Inoltre, qualsiasi modifica che dovesse intervenire nel corso dei rilevamenti sul campo, tra cui, a titolo esemplificativo, ma non esaustivo, l’incidenza effettiva, la durata delle interviste, tassi di abbandono superiori a quelli previsti, modifiche a quote e modifiche alle tempistiche per la trasmissione dei prodotti/risultati potrebbero determinare cambiamenti tanto della parte economica come di quella sulle tempistiche del preventivo.
2. **Accettazione del preventivo**. Il presente preventivo è valido per 30 giorni a decorrere dalla data in cui viene trasmesso. Il periodo di validità potrà essere eventualmente esteso, previo consenso di Dynata.
3. **Traduzioni**. In conformità con le migliori prassi del settore, il questionario sarà tradotto nella lingua correntemente parlata dai partecipanti. I costi della traduzione di Dynata sono da intendersi come costi indicativi e si riferiscono esclusivamente alla traduzione del questionario (non è inclusa la traduzione delle risposte dei partecipanti, salvo diversamente specificato). I prezzi definitivi per le traduzioni saranno calcolati sulla base di una serie di fattori, tra cui il conteggio definitivo delle parole. Nel caso in cui il Cliente decida di non tradurre lo strumento sondaggistico nella lingua locale, il Cliente conviene e riconosce che Dynata non avrà alcuna responsabilità nei confronti del Cliente per la qualità dei dati di risposta del sondaggio e il Cliente sarà tenuto ad effettuare il pagamento completo a Dynata per tutti i questionari e materiali completati in relazione a tale sondaggio.
4. **Spese per variazioni**. Siamo consapevoli che la programmazione del progetto potrebbe subire variazioni. In virtù di ciò, Dynata adotta una politica di ricalendarizzazione molto semplice: è sufficiente avvertici con un preavviso di almeno 24 ore. Dynata si adopererà al meglio delle proprie ragionevoli possibilità per accogliere e tener conto di eventuali richieste di ricalendarizzazione, in subordine alla disponibilità delle risorse richieste. Qualora non dovesse disporre delle risorse necessarie per la nuova data richiesta, Dynata proporrà al Cliente la prima data disponibile. Se non fossero disponibili le necessarie risorse per la data o le date richieste dal Cliente, Dynata provvederà a riorganizzare le

risorse in modo da poter soddisfare la richiesta per le date richieste. Tale richiesta potrebbe essere soggetta a maggiorazione. Nel caso in cui un progetto confermato venga annullato o ricalendarizzato il giorno stesso in cui era programmato, potrà essere applicata una commissione, in base al numero di risorse impegnate per la giornata in questione e/o del lavoro di preparazione già concluso.

5. **Raccolta di Dati Personali**. Il Cliente conviene e riconosce che Dynata non divulgherà o consentirà la raccolta di PII relative a partecipanti o di qualsiasi informazione di identificazione di partecipanti ("**RII**") al Cliente, ad eccezione che in situazioni di ricerca specificamente descritte, quali, ad esempio, la validazione o modellazione, consentite da e in conformità con gli standard e le pratiche professionali di settore generalmente accettati applicabili al settore di Dynata, le leggi vigenti e la politica sulla privacy di Dynata. Il Cliente riconosce e conviene espressamente che non è autorizzato e che non gli è consentito raccogliere o ricevere RII di o riguardo partecipanti di provenienza da SII a meno che: (i) Dynata fornisca il suo previo consenso scritto a tale raccolta o ricezione, consenso che può essere rifiutato, ritardato, condizionato o negato a discrezione esclusiva di Dynata, e (ii) le Parti sottoscrivano un accordo scritto distinto il cui modello sarà fornito da Dynata. Il Cliente accetta inoltre che i partecipanti non potranno essere ricontattati dal Cliente ad eccezione di come possa essere necessario per la prosecuzione di un preesistente rapporto d'affari tra il Cliente e il partecipante.

6. **Ordine minimo**. L'acquisto minimo è di 1.500 $ (o l'importo equivalente in valuta locale). I progetti con commissioni effettive inferiori a 1.500 $ verranno fatturati a 1.500 $.

7. **Ritardi nella compilazione**. Dynata potrà, a sua esclusiva discrezione, valutare se applicare una commissione per eventuali ritardi nella compilazione dei questionari. Dynata potrà applicare la maggiore tra le seguenti commissioni: 50% della tariffa completa se il partecipante interrompe o viene escluso dopo che sono trascorsi almeno 5 minuti dall'inizio dell'intervista o 100% della tariffa completa se il partecipante interrompe o viene escluso dopo che è trascorsa almeno la metà del tempo programmato per l'intervista secondo il preventivo.

8. **Pagamenti**. Tutti i prezzi sono da intendersi al netto di eventuali imposte, mentre i termini di pagamento sono a 30 gg. netti. Salvo diversamente disciplinato dalla normativa locale, in caso di ritardi agli importi dovuti e non contestati saranno maggiorati a un interesse pari al due per cento (2%) al mese. Le Parti riconoscono e convengono che le informazioni relative al progetto e contenute in questo preventivo possono essere soggette a modifica nel corso del progetto. La fattura definitiva trasmessa al Cliente deve rispecchiare i servizi effettivamente erogati e comprendere tutte le modifiche e commissioni di cui sopra.

9. Dynata offre servizi professionali di programmazione e hosting e interviste telefoniche in modalità CATI (Computer-Assisted Telephone Interviewing) attraverso le proprie società interamente controllate e/o attraverso la propria società capogruppo o le consociate interamente controllate. Per tutti i servizi eseguiti da Dynata ai sensi del presente, Dynata stabilirà a propria discrezione quale sede Dynata fornirà tali servizi. Qualora il Cliente, direttamente o tramite agenti intermediari, scelga di programmare e/o ospitare un progetto online, lo stesso conviene quanto segue: (i) che verranno utilizzati link sicuri criptati con cifratura token ("**Link sicuri**") e, qualora il Cliente non riesca ad utilizzare il Link sicuro, Dynata potrà chiedere il rimborso al Cliente per qualsiasi premio/incentivo corrisposto al partecipante fatto in relazione a sondaggi fraudolenti completati; (ii) i link dei sondaggi consentiranno ai partecipanti idonei di completare il sondaggio corretto; (iii) verranno utilizzati server stabili di capacità adeguata, un sistema di interviste interattivo in grado di collegare gli ID dei partecipanti forniti da Dynata ai questionari e poi di nuovo all'URL di Dynata, e un'interfaccia grafica compatibile con Dynata (con background JPG); e (iv) nessun contenuto di sondaggi del cliente o ospitati da intermediari del cliente contiene o conterrà virus, malware o script sospetti. Qualora Dynata ritenga che vi sia qualsiasi Malware reale o presunto, Dynata ha il diritto di sospendere il progetto fino alla risoluzione dei problemi.

10. **Riservatezza**. Le parti sono tenute a mantenere la riservatezza di ogni informazione e materiale che ricevono dall'altra parte che siano legati all'attività aziendale di tale parte o ritenuti proprietari e riservati da suddetta parte che li divulga. Resta inteso che tale clausola di riservatezza non include informazioni che: (i) siano attualmente o in futuro di dominio pubblico non a causa di negligenza della parte a cui le informazioni riservate siano state fornite; (ii) prima della divulgazione ai sensi del presente, siano di proprietà e nel legittimo possesso della parte a cui siano state fornite le informazioni riservate; (iii) successivamente alla divulgazione ai sensi del presente, siano legittimamente ricevute da una parte terza senza restrizione alcuna in merito ad ulteriore divulgazione; (iv) siano state sviluppate indipendentemente dalla parte ricevente senza fare affidamento sulle informazioni riservate della parte divulgante. Ciascuna parte quivi si impegna a non rivelare le informazioni riservate dell'altra parte a qualsivoglia parte terza senza

la preventiva autorizzazione scritta dell'altra. Nel caso in cui Dynata venga contattata da un membro o da membri della stampa in cerca di commenti riguardo a presunte violazioni delle leggi applicabili derivanti dal contenuto di un sondaggio fornito da o per conto del Cliente, Dynata può, previo avviso al Cliente, fornire il nome e le informazioni di contatto del Cliente a tale parte o parti richiedenti senza dar luogo a una violazione della presente sezione.

11. **Proprietà**

(i) Per tutti i servizi di raccolta di dati svolti da Dynata e/o per qualsivoglia sondaggio ospitato da Dynata, le parti concordano che Dynata e il Cliente debbano avere la proprietà congiunta di tutti i diritti, titoli e interessi relativi alle risposte nei sondaggi raccolte da Dynata e/o di qualsivoglia dato aggiuntivo raccolto dai partecipanti tramite la piattaforma di Dynata.

(ii) Fatta eccezione per campioni e questionari forniti dal Cliente, i panel, i partecipanti, le comunità partecipanti; le informazioni di identificazione di partecipanti, tecniche, tecnologie, metodologie, concetti, invenzioni, suggerimenti, idee creative, piani, disegni, progetti, disegni computerizzati, modelli o sistemi, prototipi, modelli di ricerca, modelli di questionari (salvo che forniti dal Cliente), metodi di elaborazione di domande, sistemi di analisi, software, tecnologie e/o programmi informatici forniti e/o utilizzati da Dynata in relazione al presente progetto/ordine, schede di tabulazione, nastri informatici, dischetti e qualsiasi altro formato di registro di dati, sono e rimarranno di proprietà esclusiva di Dynata, LLC e in nessun caso il Cliente potrà ottenere o ricevere alcun diritto, titolo e/o licenza su e/o in relazione ad uno qualsiasi di quanto precede.

(iii) Fermo restando quanto diversamente previsto nei presenti termini, in nessun caso i prodotti/risultati attesi (prodotto del lavoro di proprietà del Cliente) includeranno PII di partecipanti della ricerca di provenienza da Dynata, anche qualora tali PII siano incluse nelle risposte, nei dati e/o nelle relazioni/nei registri del sondaggio.

12. **Utilizzo dei tag; Dati Aggregati**.

(i) Salvo il previo consenso espresso scritto da parte di Dynata, il cui consenso potrebbe essere condizionato, trattenuto, o negato a mera discrezione di Dynata, il Cliente quivi accetta espressamente che il Cliente, direttamente o indirettamente, non:

(a) scriverà, imposterà o rilascerà alcun cookie, pixel, o tecnologia correlata o simile sui computer o dispositivi di partecipanti individuati tramite Dynata ("**Partecipanti individuati da Dynata**") al fine di tracciare l'attività dei partecipanti su Internet, su sondaggi multipli, su piattaforme multiple, o su dispositivi o computer multipli; o

(b) accederà a un computer o dispositivo di qualsivoglia Partecipante individuato da Dynata per leggere qualsivoglia cookie o altra tecnologia simile o correlata, o per accedere e/o raccogliere informazioni archiviate su o all'interno del dispositivo o computer.

(ii) Salvo (a) il previo consenso scritto da parte di Dynata, il cui consenso potrebbe essere condizionato, trattenuto, o negato a mera discrezione di Dynata, o (b) in relazione a un sondaggio di tracciabilità ai sensi di o in relazione a un progetto; il Cliente non potrà utilizzare qualsivoglia dato a livello del singolo partecipante da due o più progetti di sondaggio o studi per costruire, creare, sviluppare, aggiornare, potenziare o implementare qualsivoglia file, elenco o database, sia per il beneficio del cliente che di qualsivoglia parte terza.

13. **Disposizioni varie**.

(i) **Obblighi del Cliente**. Il Cliente si impegna ad osservare tutte le norme e i regolamenti applicabili relativi al progetto di ricerca oggetto del presente preventivo, la dichiarazione di attività (statement of work) o qualsiasi altro documento specifico relativo al progetto fornito da Dynata e il Cliente, da parte sua, conferma che l'intervista in questione ha come finalità principale una ricerca di mercato. Il Cliente accetta inoltre che (a) qualora fornisca dati personali a Dynata, inclusi ma non limitatamente a, indirizzi di posta elettronica e/o numeri di telefono a Dynata, il cliente o il cliente del medesimo hanno il permesso da parte del soggetto(i) di condividere tali informazioni con Dynata al fine di condurre sondaggi online e/o ricerche di mercato; e nell'ipotesi in cui il Cliente fornisca un campione telefonico o online a Dynata che provengano da una parte terza, il Cliente dichiara e garantisce che tale parte terza acconsente alla condivisione di tale campione con Dynata e consente all'utilizzo del campione da parte di Dynata per conto del Cliente come stabilito nell'Accordo e/o Dichiarazione di attività (Statement of Work, SOW); e (b) qualora il Cliente utilizzi Dynata per raccogliere dati in relazione a un sondaggio

online o telefonico e tale indagine includa partecipanti residenti in Russia, tale sondaggio non dovrà richiedere informazioni di identificazione personale (PII) da tali partecipanti.

(ii) **Indennizzo da parte del Cliente**. Il Cliente dovrà manlevare, difendere e tenere indenne Dynata, nonché le sue società capogruppo, le consociate e affiliate e i loro rispettivi soci, amministratori, azionisti, direttori, dirigenti, dipendenti e agenti da e nei confronti di tutte le responsabilità, perdite, danni, pretese, azione legali, lodi, sentenze, oneri e costi (ivi comprese le ragionevoli spese legali e gli onorari di avvocati) che Dynata dovesse sostenere, a causa di o in relazione a: (i) violazione da parte del Cliente di normative, codici, regolamenti e requisiti applicabili; (ii) atti di negligenza, omissioni, dolo del Cliente e/o violazioni da parte dello stesso dei propri obblighi qui definiti; e/o (iii) qualsiasi prodotto o servizio fornito o prestato da o per conto del Cliente ai partecipanti al sondaggio per il suo uso o prova relativamente al progetto.

(iii) **Limitazione di responsabilità**. Nessuna delle Parti sarà ritenuta responsabile nei confronti dell'altra per qualunque danno indiretto, incidentale, esemplare, speciale o conseguente (ivi compreso a titolo esemplificativo, ma in via non limitativa, l'eventuale perdita di profitti), indipendentemente dal fatto che una Parte sia informata della sua eventualità. Con la presente il Cliente esonera espressamente Dynata da qualsiasi pretesa o responsabilità, in toto o in parte, relativamente a: (i) l'uso, l'interpretazione e/o l'affidamento da parte del Cliente dei dati o delle risposte ricevuti o raccolti in connessione ai servizi di Dynata, e (ii) azioni, attività o comportamenti dei partecipanti al sondaggio. Fatti salvi gli obblighi di indennizzo del Cliente e le violazioni da parte del Cliente delle normative applicabili, il valore complessivo delle responsabilità di ciascuna delle Parti sarà limitato agli importi dovuti dal Cliente (che possono eventualmente comprendere anche eventuali interessi su pagamenti scaduti) in relazione alla Dichiarazione di attività a cui attenga la responsabilità o per cui venga pretesa l'indennità.

(iv) **Esclusione di responsabilità**. IL CLIENTE RICONOSCE E ACCETTA CHE Dynata FORNISCE I PROPRI SERVIZI "COME TALI". SALVO ALTRIMENTI SPECIFICATO NEL PRESENTE DOCUMENTO, CON LA PRESENTE Dynata DECLINA OGNI GARANZIA, IMPLICITA O ESPLICITA, IN RELAZIONE AI SERVIZI E AD EVENTUALI PRODOTTI/DOCUMENTI FINALI TRASME Dynata AI SENSI DEL PRESENTE, COMPRESI, A TITOLO ESEMPLIFICATIVO, MA IN VIA NON LIMITATIVA, GARANZIE IMPLICITE DI NON VIOLAZIONE ED EVENTUALI GARANZIE IMPLICITE DERIVANTI DA UNA PRESTAZIONE, TRATTATIVA O USO COMMERCIALE. NELLA MISURA IN CUI, IN VIRTÙ DI NORMATIVE APPLICABILI, Dynata NON PUÒ DECLINARE UNA QUALUNQUE GARANZIA, IL CAMPO DI APPLICAZIONE E LA DURATA DELLA STESSA SARANNO LIMITATE AL MINIMO CONSENTITO AI SENSI DELLA NORMATIVA APPLICABILE.

(v) **Legge applicabile**. Questo Ordine/Progetto e i presenti termini saranno disciplinati da, saranno soggetti a e saranno interpretati e fatti rispettare in conformità alle leggi dell'Inghilterra e del Galles e i tribunali competenti dell'Inghilterra e del Galles saranno il foro competente e avranno giurisdizione esclusiva per la risoluzione di qualsiasi eventuale controversia in relazione al presente.

(vi) **Cessione/Subappalto**. Dynata si riserva il diritto, senza fornire preavviso al Cliente e senza ottenere il consenso dello stesso, di cedere in tutto o in parte e/o a subappaltare una o più parti dell'esecuzione dei servizi/del progetto a qualsiasi società affiliata e/o a qualsiasi terza parte.

Le Parti convengono che i termini e le condizioni legali contenuti in o su qualsiasi documento o modulo presentato dal Cliente sono nulli, invalidi e privi di efficacia o effetto.
